# *Akkermansia muciniphila* regulates the gut microenvironment and alleviates periodontal inflammation in mice with periodontitis

**DOI:** 10.3389/fmicb.2025.1643691

**Published:** 2025-09-17

**Authors:** Shumin Zhang, Ting Zhang, Yiwen Zhang, Chuanjin Ye, Litong Mu, Qinghui He, Tianxiang Huang, Guowei Wang, Yanan Li, Sijing Xie, Xuna Tang

**Affiliations:** 1 Nanjing Stomatological Hospital, Affiliated Hospital of Medical School, Institute of Stomatology, Nanjing University, Nanjing, China; 2 School of Food Science and Pharmaceutical Engineering, Nanjing Normal University, Nanjing, China

**Keywords:** periodontitis, *Akkermansia muciniphila*, gut microbiota, oral-gut axis, fecal microbiota transplantation

## Abstract

**Objective:**

*Akkermansia muciniphila* (*A. muciniphila*) is an emerging gut commensal known for its roles in host metabolism and immune modulation. While its involvement in metabolic and inflammatory disorders is well characterized, its potential association with oral diseases such as periodontitis remains poorly understood. This study aimed to explore whether modulation of the gut microbiota via fecal microbiota transplantation (FMT) from periodontally healthy donors could influence the abundance of *A. muciniphila* and contribute to the alleviation of periodontitis.

**Methods:**

Fecal samples were collected from human donors, including periodontally healthy individuals (H group, *n* = 16), untreated patients with severe periodontitis (P group, *n* = 12), and the same patients at two weeks (P2W) and three months (P3M) after periodontal therapy. Quantitative PCR was used to assess *A. muciniphila* abundance in these human samples. A germ-free mouse model of periodontitis was then established, and the mice received FMT using samples from human donor groups (P-PBS, P-H, and P-P). Gut microbiota composition, periodontal inflammation, gut barrier proteins (MUC2, ZO-1), and inflammatory cytokines (IL-6, TNF-α) were evaluated in the mice.

**Results:**

Compared to groups H, P2W, and P3M, the abundance of *A. muciniphila* in the gut was significantly lower in patients with severe periodontitis, but it was increased after periodontal therapy. In mice, FMT from healthy donors (P-H group) significantly enriched *A. muciniphila*, improved expression of gut barrier proteins, reduced inflammatory cytokine levels, and alleviated periodontal inflammation compared to other groups.

**Conclusion:**

These findings suggest a previously underrecognized link between gut microbial composition particularly *A. muciniphila* and periodontal health. Targeting the gut microbiota via FMT may represent a novel strategy for modulating systemic and oral inflammation and supporting the prevention or adjunctive treatment of periodontitis.

## 1 Introduction

According to the Global Burden of Disease Study (GBD) 2021 (GBD 2021 Oral Disorders Collaborators, 2025), the number of individuals affected by severe periodontitis has increased by 91.5% worldwide, highlighting its significant impact on global public health. The age-standardized global prevalence has reached approximately 12,500 cases per 100,000 population, underscoring the urgent need for effective prevention and treatment strategies. An expanding body of evidence suggests that periodontitis may influence systemic health through the oral-gut axis (Kunath et al., 2024), contributing to conditions such as inflammatory bowel disease (IBD), diabetes mellitus, and Alzheimer’s disease. Oral microbiota can reach the gut either hematogenously or via the gastrointestinal tract, leading to dysbiosis through ectopic colonization, reduced alpha diversity, and disruption of the intestinal barrier, ultimately promoting intestinal inflammation (Elghannam et al., 2024). Patients with severe periodontitis harbor large quantities of pathogenic bacteria such as *Porphyromonas gingivalis* and *Actinomyces* species (Sulaiman et al., 2024). Compared with healthy controls, individuals with severe periodontitis exhibit increased abundance of *Bacteroidetes* and *Firmicutes* in their fecal samples, while *Lactobacillus* is the only genus found in higher abundance in healthy individuals. Periodontal treatment significantly reduces the abundance of periodontal pathogens and *Bacteroides*, restoring microbiota composition to levels comparable to healthy controls (Baima et al., 2024). These findings suggest that periodontitis can affect gut microbial composition via salivary transmission. However, the reciprocal relationship—how gut microbiota may induce or modulate periodontitis—remains largely unexplored.

Fecal microbiota transplantation (FMT), the process of transferring gut microbiota from healthy donors to recipients, has emerged as a promising strategy for modulating dysbiotic microbiomes in various diseases (Wu et al., 2023). Originally developed for treating recurrent *Clostridioides difficile* infection, FMT has since demonstrated potential therapeutic benefits in metabolic disorders, inflammatory bowel diseases, and even neuroimmune conditions by restoring gut microbial diversity and host immune balance (Hanssen et al., 2021). Given the bidirectional interaction between the oral cavity and gut, recent studies have begun to explore the influence of gut microbiota on oral inflammatory diseases, including periodontitis. However, whether FMT from periodontally healthy donors could reshape gut microbial profiles and alleviate periodontal inflammation remains unclear, and the reciprocal relationship—how gut microbiota may induce or modulate periodontitis—remains largely unexplored. Notably, colitis has been reported to exacerbate periodontal inflammation (Xu et al., 2025). Mendelian randomization studies (Song J. et al., 2023) have identified 16 gut bacterial taxa associated with periodontitis and tooth loss, including five species within the *Lactobacillaceae* family linked to increased periodontal disease risk.

*Akkermansia muciniphila* is a Gram-negative, anaerobic bacterium of the phylum Verrucomicrobia, named for its ability to degrade mucin and primarily colonize the intestinal mucus layer (Guo et al., 2017). It plays a pivotal role in maintaining intestinal barrier function, modulating immune responses, and regulating metabolic homeostasis (Ioannou et al., 2025). Through mucin degradation, *A. muciniphila* produces short-chain fatty acids (SCFAs), which serve as an energy source for intestinal epithelial cells and help regulate gut permeability (Chelakkot et al., 2018). Moreover, *A. muciniphila* has been shown to suppress inflammatory responses, improve insulin sensitivity, and exhibit therapeutic potential in metabolic disorders such as obesity and type 2 diabetes (Yan et al., 2021). Emerging evidence suggests that *A. muciniphila* may also influence the onset and progression of periodontitis through several mechanisms (Anderson et al., 2024). It can inhibit the growth of oral pathogens like *P. gingivalis* and *Fusobacterium nucleatum* (Mulhall et al., 2022; Huck et al., 2020; Song B. et al., 2023; Hu et al., 2025), thereby contributing to the maintenance of oral microbial homeostasis. Metabolites produced by *A. muciniphila*, particularly SCFAs, may suppress the proliferation of periodontopathogens by modulating local pH and nutrient availability (Elzinga et al., 2024). Additionally, *A. muciniphila* may influence periodontal inflammation by promoting regulatory T cell differentiation and attenuating excessive immune responses (Ansaldo et al., 2019). These immunomodulatory effects could help limit tissue damage in periodontal disease. Furthermore, its ability to enhance epithelial barrier function may offer protection against microbial invasion and dissemination (Zhang et al., 2019). Clinical evidence indicates that *A. muciniphila* abundance is significantly reduced in the oral cavity of periodontitis patients, suggesting a protective role in periodontal health.

In this study, we established a murine model of periodontitis to assess changes in the abundance of *A. muciniphila* in the gut. We further investigated how FMT from different sources influences gut microbial composition and the progression of periodontitis. Our aim is to elucidate the role of gut microbiota in the oral-gut axis and to highlight the potential of *A. muciniphila* as an adjunctive therapeutic agent in the management of periodontitis.

## 2 Materials and methods

### 2.1 Human fecal sample collection and processing

The sample size for human participants was determined based on previous studies (Bao et al., 2022) evaluating gut microbial changes associated with periodontitis. Assuming a moderate effect size (Cohen’s *d* = 0.8), a significance level (*α*) of 0.05, and power of 0.8, a minimum of 12 individuals per group was estimated to detect statistically significant differences in microbiota composition. Therefore, fecal samples were collected from 16 periodontally healthy individuals and 12 patients with severe periodontitis at the Affiliated Stomatological Hospital of Nanjing University School of Medicine, with approval from the Ethics Committee of the Nanjing University School of Medicine (NJSH-2022NL-43). The diagnosis of periodontitis with severe periodontitis was based on the 2017 World Workshop classification system (Papapanou et al., 2018). All patients included in this study were classified as having Stage III or IV periodontitis, generalized extent, with PD ≥6 mm and radiographic bone loss (RBL) extending to the middle or apical third of the root. Diagnosis and classification were confirmed by a calibrated periodontist. The inclusion and exclusion criteria for volunteers is provided in the Supplementary material. All human participants provided written informed consent prior to sample collection. Noted, twelve patients diagnosed with severe periodontitis were enrolled and sampled at three time points: prior to treatment (P group), two weeks after non-surgical periodontal therapy (P2W group), and three months post-treatment (P3M group). All patients received standard non-surgical periodontal therapy, including supragingival and subgingival scaling and root planning performed by a certified periodontist. No systemic antibiotics were prescribed during treatment. Fresh fecal samples were collected using sterile collection kits. Approximately 3–5 g of the middle portion of the stool was placed into sterile tubes, mixed with 20% sterile glycerol, rapidly frozen in liquid nitrogen, and stored at −80 °C until further use. A portion of each sample was used for quantitative PCR. Another portion was thawed on ice and processed for fecal microbiota transplantation. Briefly, 200 mg of feces was diluted in 2 mL sterile PBS, vortexed for 5 min, filtered, and centrifuged at 600 × g for 5 min to remove insoluble material.

### 2.2 Mice and study design

Eight-week-old male C57BL/6J mice were approved by the Animal Welfare and Ethics Review Committee of Jiangsu Aniphebio Co., Ltd. (JSAB24021M).

This study consisted of two parts:

#### 2.2.1 Study 1: alteration of gut microbial structure in healthy and periodontitis mice

To investigate the gut microbial changes associated with periodontitis, a mouse model was established using ligature placement. Sixteen mice were randomly assigned to two groups: healthy control group (H, *n* = 8) and periodontitis group (P, *n* = 8). In the periodontitis group, bilateral ligatures were placed around the maxillary first molars. After two weeks, the periodontitis model was successfully established, and fecal samples were collected for microbial analysis.

#### 2.2.2 Study 2: FMT in periodontitis mice

**Figure 1:**
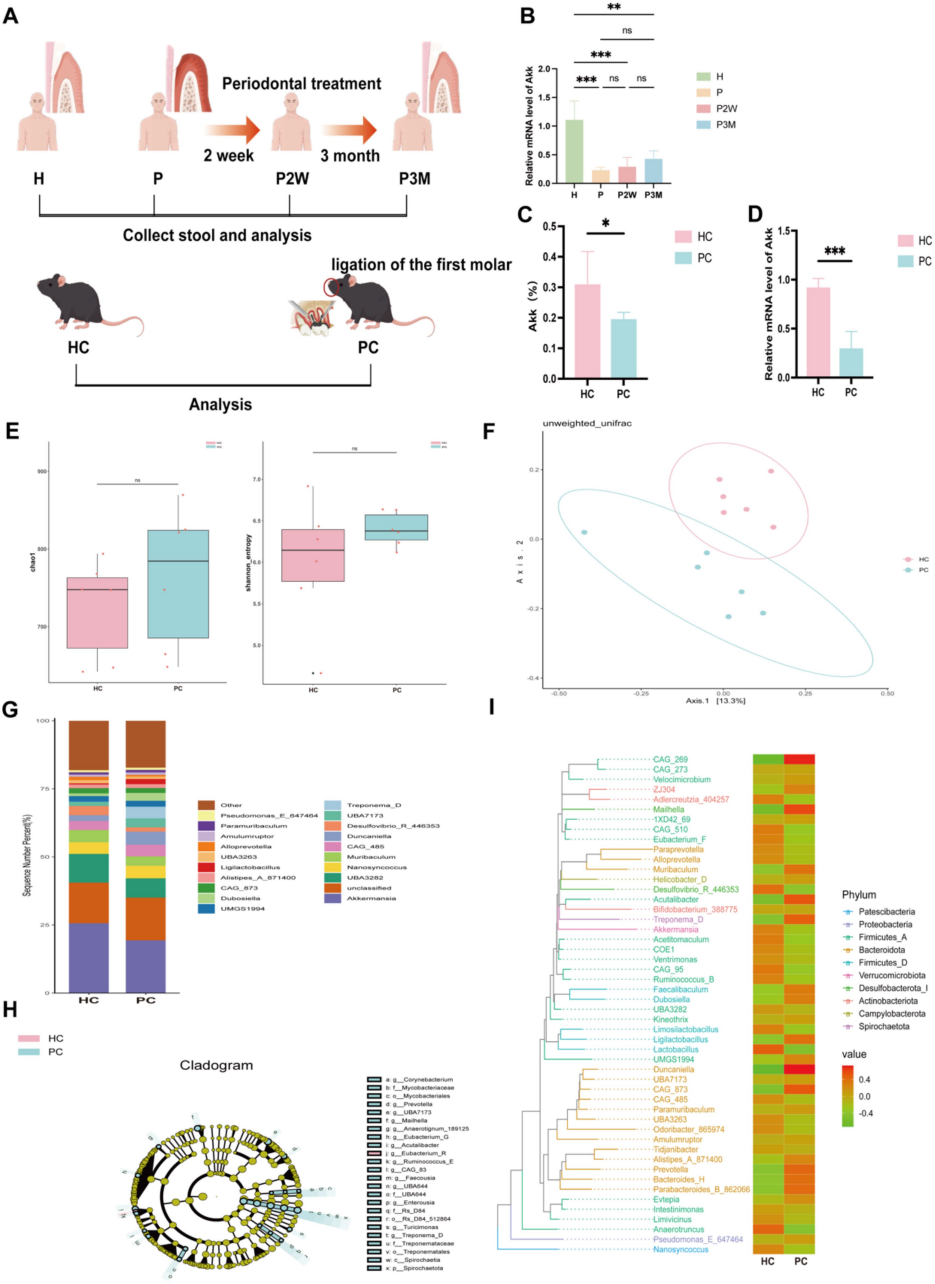
Gut microbiota distribution in healthy and periodontitis mice. **(A)** Experimental design flowchart (experimental grouping and procedures are detailed in the materials and methods section). **(B)** Differences in the expression levels of *Akkermansia muciniphila* in fecal samples from healthy individuals (H), patients with severe periodontitis (P), and patients two weeks (P2W) and three months (P3M) post-treatment. Data are presented as mean ± SD. Statistical significance was determined by one-way ANOVA. ^*^p < 0.05, ^**^p < 0.01, ^***^p < 0.001, and ns: not significant. **(C)** Differences in the abundance of *A. muciniphila* between healthy (HC) and periodontitis mice (PC) as analyzed by 16S rRNA sequencing. **(D)** Differences in the expression levels of *A. muciniphila* in the gut of HC and PC. **(E)** Alpha diversity index statistics, including Chao1 and Shannon indices, reflecting species richness. **(F)** *β*-diversity index statistical analysis based on PCoA. **(G)** Top 20 genera bar chart at the genus classification level. **(H)** LEfSe analysis cladogram. **(I)** Phylogenetic tree heatmap.

**Figure 2:**
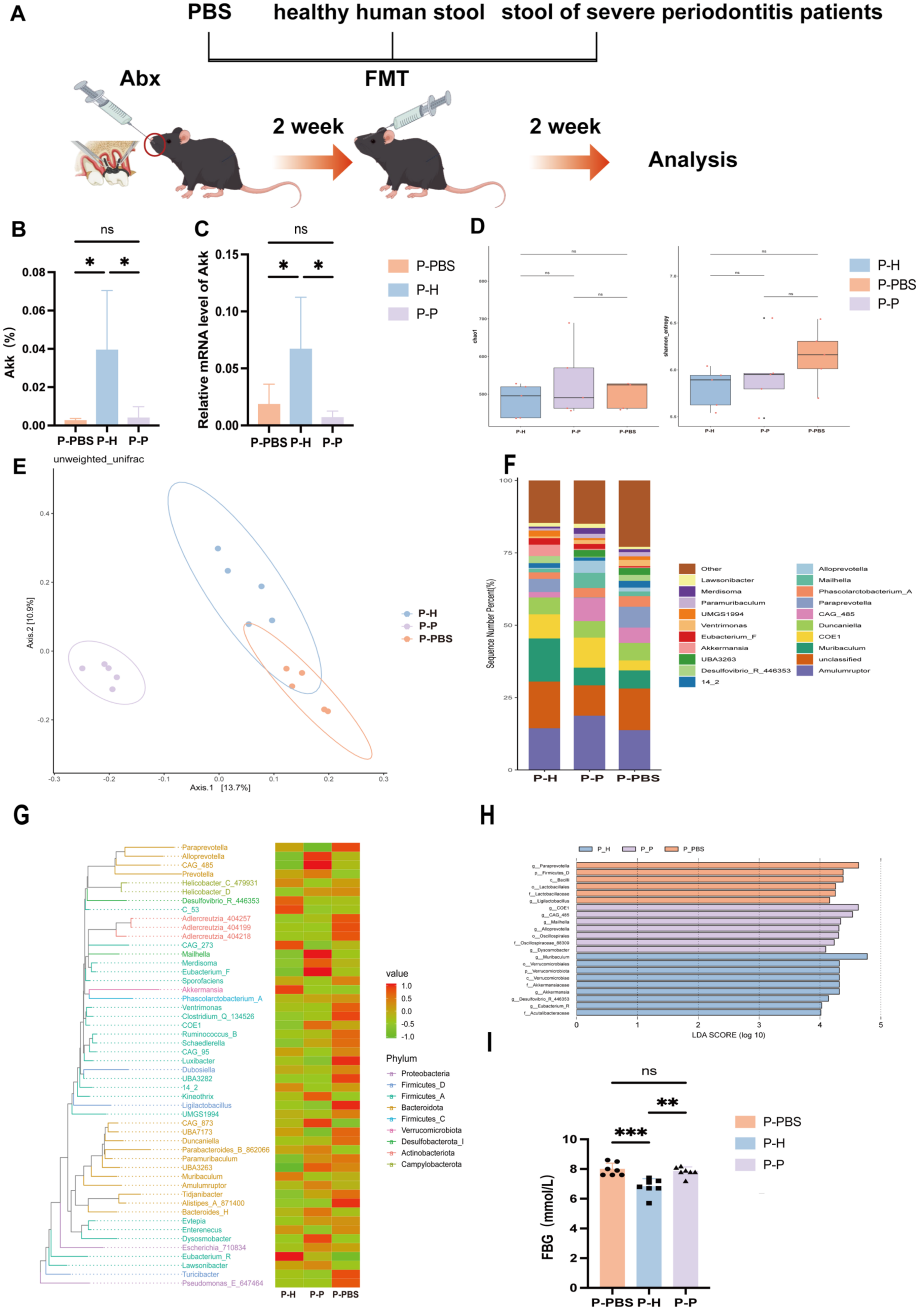
Changes in gut microbiota of periodontitis mice following PBS, healthy human fecal microbiota, and severe periodontitis patient fecal microbiota gavage treatments. **(A)** Experimental design flowchart. **(B)** Differences in *A. muciniphila* levels in the gut of periodontitis mice after different gavage treatments, as analyzed by 16S rRNA sequencing. Data are presented as mean ± SD. Statistical significance was determined by one-way ANOVA. ^*^p < 0.05, ^**^p < 0.01, ^***^p < 0.001, and ns: not significant. **(C)** Differences in *A. muciniphila* expression levels in the gut. **(D)** Alpha diversity index statistics, including Chao1 and Shannon indices. **(E)** β-diversity index statistical analysis based on PCoA. **(F)** Relative distribution of genera bar chart (top 20 species by relative abundance). **(G)** phylogenetic heatmap. **(H)** LEfSe analysis cladogram. **(I)** Fasting blood glucose levels.

To assess the impact of gut microbiota modulation on periodontal inflammation, 24 periodontitis mice were divided equally into three treatment groups (*n* = 8/group): a P-PBS group receiving phosphate-buffered saline (PBS), a P-H group receiving fecal microbiota transplants (FMT) from periodontally healthy human donors, and a P-P group receiving FMT from patients with severe periodontitis. Before transplantation, all mice underwent a two-week native gut microbiota depletion protocol using a broad-spectrum antibiotic cocktail (ampicillin 1 mg/mL, vancomycin 0.5 mg/mL, neomycin 1 mg/mL, metronidazole 1 mg/mL) administered in their drinking water (Tan et al., 2022). FMT was subsequently performed via oral gavage, with each mouse receiving 200 mg of fecal suspension per administration, 2–3 times weekly over two consecutive weeks, as outlined in Figures 1A, 2A.

### 2.3 16S rRNA gene sequencing analysis

Genomic DNA was extracted from mouse fecal samples using the CTAB method (Nobleryder, China). According to the manufacturer’s instructions, the V4 region of the 16S rRNA gene was amplified using a PCR kit (New England Biolabs, United States), with primers 515F (5′-GTGCCAGCMGCCGCGGTAA-3′) and 806R (5′-GGACTACHVGGGTWTCTAAT-3′). After PCR amplification, the products were detected by agarose gel electrophoresis and then purified using a Universal DNA Purification Kit (Tiangen, China). Gene libraries were constructed using a library kit (E7370L, Illumina, United States) and sequenced on a Novaseq 6000 PE250 platform. Raw sequencing data were processed using QIIME2 (2022.2). The DADA2 plugin was used for quality filtering, denoising, chimera removal, and feature table construction. Amplicon sequence variants (ASVs) were taxonomically classified by comparing representative sequences against the SILVA 138 database. Microbial community diversity was assessed using alpha diversity indices (Shannon, Chao1) and beta diversity (Bray–Curtis dissimilarity). Principal coordinate analysis (PCoA) was performed to visualize inter-group differences. Linear discriminant analysis Effect Size (LEfSe) was used to identify differentially abundant taxa among experimental groups.

### 2.4 Micro-CT analysis

The maxillary bone specimens of mice were fixed in 4% paraformaldehyde for 24 h, trimmed, and excess tissue was removed. The specimens were then washed three times with PBS, and scanned using Micro-CT (Viva CT40, SCANO). The scanning parameters were set to a resolution of 10 μm, voltage of 70 kV, and current of 114 mA.

### 2.5 Detection of *Akkermansia muciniphila* in fecal samples

Bacterial DNA was extracted from human and mouse fecal samples using the TIANamp Stool DNA Kit (Tiangen Biotech, China). Fluorescent quantitative PCR was then performed to detect the relative expression of *A. muciniphila* in the fecal microbiota. The relative gene expression was calculated using the 2^−ΔΔCT^ method. The primer sequences for species-level detection were as follows: *27*F, 5′-AGAGTTTGATCCTGGCTCAG-3′; *1492*R, 5′-TACGGCTACCTTGTTACGACTT-3′; *A. muciniphila* F, 5′-CAGCACGTGAAGGTGGGGAC-3′; R, 5′-CCTTGCGGTTGGCTTCAGAT-3′.

### 2.6 Detection of colonic tissue-related proteins and inflammatory cytokine levels

Total RNA was extracted from mouse colon tissue using the TRIzol method. cDNA was synthesized using a reverse transcription kit (Novozyme, China). Fluorescent quantitative PCR was performed with cDNA as the template to detect the relative expression levels of inflammatory cytokines such as TNF-α, IL-10, tight junction proteins like CLAUDIN15, JAM3, and MUC2 mucin in colon tissue. The relative gene expression levels of RNA were calculated using the 2^−ΔΔCT^ method. The primer sequences for species-level detection were as following:

*GADPH*F, 5′-GGTTGTCTCCTGCGACTTCA-3′; R, 5′-TGGTCCAGGGTTTCTTACTCC-3′; TNF-*α*F, 5′-GGCGTGTTCATCCGTTCTC-3′; R, 5′-CTTCAGCGTCTCGTGTGTTTCT-3′; IL-*6*F, 5′-CGCCCCACACAGACAGCCAC-3′; R, 5′-AGCTTCGTCAGCAGGCTGGC-3′; OCCLUDINF, 5′-TGGCGGATATACAGACCCAA-3′; R, 5′-CGATCGTGGCAATAAACACC-3′; JAM*3*F, 5′-GAGACTCAGCCCTTTATCGC-3′; R, 5′-CCTTCGGCACTCTACAGACA-3′; CLAUDIN*15*F, 5′-CTGCTAACCTGAAAGGGCA-3′; R, 5′-GGGACTGCTGGAATGAGACC-3′; ZO-*1*F, 5′-GCCGCTAAGAGCACAGCAA-3′; R, 5′-GCCCTCCTTTTAACACATCAGA-3′; MUC*2*F, 5′-TGCCCACCTCCTCAAAGAC-3′; R, 5′-GTAGTTTCCGTTGGAACAGTGAA-3′.

### 2.7 Histological and immunohistochemical analysis

Hematoxylin and eosin (HE) staining was used to evaluate gingival and alveolar bone inflammation, as well as colonic crypt morphology. Immunohistochemical staining was conducted to detect IL-1β in periodontal tissue and ZO-1 and MUC2 in colonic tissue, using antibodies from Affinity Biosciences (USA) and Servicebio (China), respectively. Positive staining areas were quantified using ImageJ software.

### 2.8 Statistical methods

All data are presented as the mean ± standard deviation. Statistical analysis was performed using GraphPad Prism 10.0. For comparisons between two groups, a *t*-test was used. For more than three groups, one-way ANOVA with Bonferroni post-hoc tests was applied. Correlation analysis was conducted using Pearson’s correlation algorithm. *p*-values are considered significant as follows: ^*^p < 0.05, ^**^p < 0.01, ^***^p < 0.001, and ns: not significant.

## 3 Results

### 3.1 The abundance of *Akkermansia muciniphila* in the gut microbiota was higher in healthy mice compared to periodontitis-induced mice

We analyzed the expression levels of *Akkermansia muciniphila* in fecal samples from healthy individuals and periodontitis patients at different treatment phases. qPCR analysis (Figure 1B) showed that *A. muciniphila* levels were significantly reduced in untreated patients with periodontitis compared to healthy individuals. Although no statistically significant differences were observed among the periodontitis groups, a gradual increasing trend was noted after periodontal therapy (P2W and P3M), indicating a potential recovery trajectory. To eliminate the confounding effects of individual lifestyles and dietary habits, I established a periodontitis mouse model and analyzed differences in gut luminal contents between healthy mice and periodontitis mice.16S rRNA sequencing of fecal samples (Figure 1A) showed that *A. muciniphila* abundance was significantly higher in HC mice compared to PC mice (Figures 1C,D). Although *α*-diversity did not differ significantly (Figure 1E), β-diversity was notably altered, as shown by PCoA based on unweighted UniFrac distances (Figure 1F), indicating distinct microbial communities between groups. At the genus level (Figure 1G), PC mice exhibited increased *Duncaniella* and *Treponema-D* and decreased *Akkermansia* and *Muribaculum*. *Treponema-D*, a known oral pathogen, was elevated in the gut of PC mice, suggesting possible microbial translocation. Additionally, PC mice showed higher *Bacteroidota* and lower *Firmicutes* and *Verrucomicrobiota* levels. Cladogram (Figure 1H) and phylogenetic analysis (Figure 1I) confirmed enrichment of *A. muciniphila*, *Lactobacillus*, and *Eubacterium* in HC mice. Together, these results suggest that periodontitis is associated with reduced *A. muciniphila* and gut dysbiosis.

### 3.2 Periodontitis induces alveolar bone loss and alters intestinal barrier function in mice

**Figure 3:**
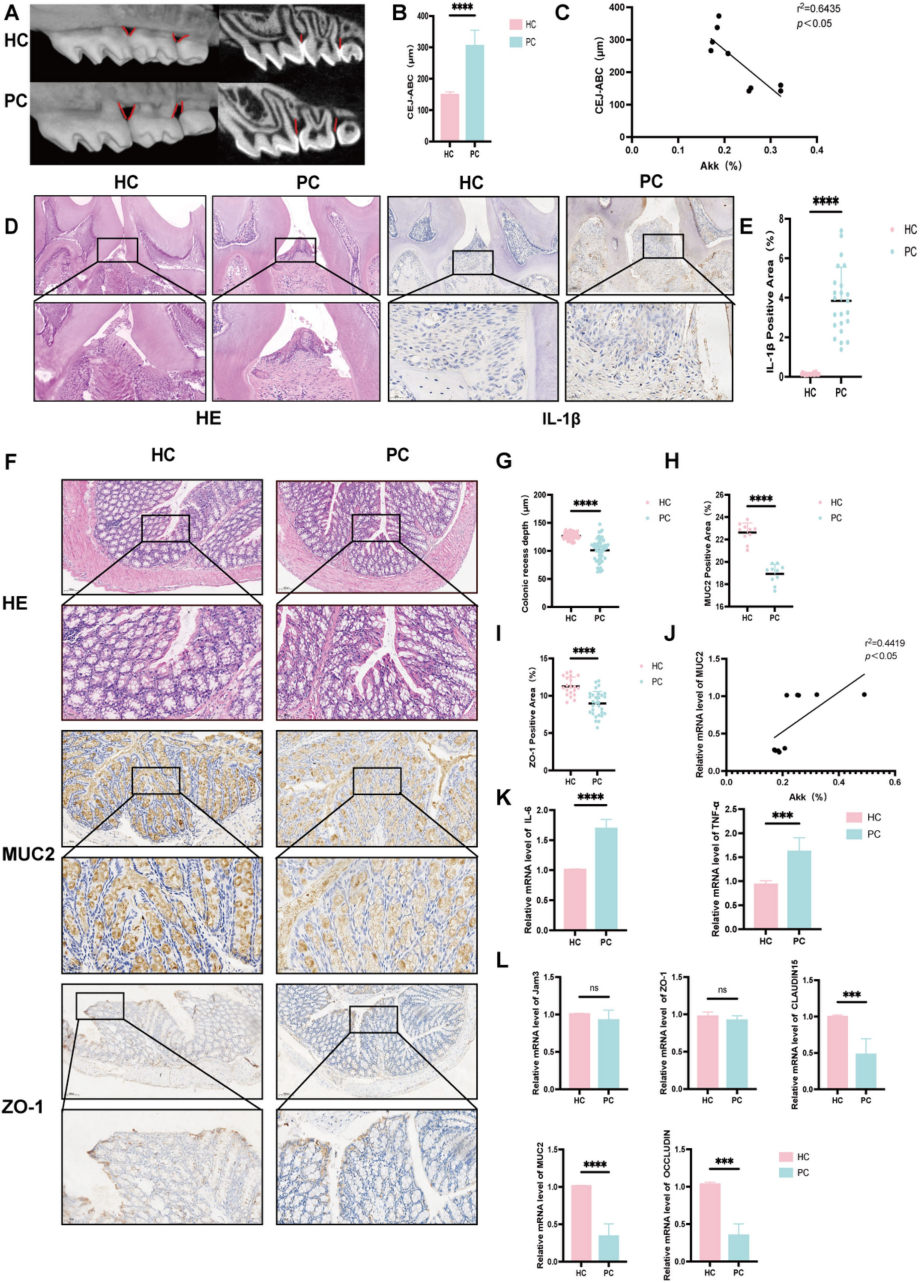
Expression levels of proteins, inflammatory factors, etc., in the gut of healthy and periodontitis mice. **(A)** Micro-CT three-dimensional reconstruction images of healthy (HC) and periodontitis mice (PC). **(B)** Distance between the cementoenamel junction (CEJ) and alveolar bone crest (ABC), representing the degree of alveolar bone resorption. **(C)** Correlation analysis between *A. muciniphila* levels and the CEJ-ABC distance. **(D)** HE and IL-1β staining sections of maxillary bone tissue (scale bars: 50 μm and 25 μm, respectively). **(E)** Percentage of IL-1β positive expression. **(F)** HE, MUC2, and ZO-1 staining sections of colonic tissue. **(G)** Colonic crypt depth. **(H)** Percentage of MUC2 positive expression in the colon. **(I)** Correlation analysis between *A. muciniphila* levels and MUC2 expression in the colon. **(J)** Percentage of ZO-1 positive expression in the colon. **(K)** mRNA expression levels of IL-6 and TNF-α. **(L)** mRNA expression levels of ZO-1, JAM3, CLAUDIN15, OCCLUDIN, and MUC2. Data are presented as mean ± SD. Statistical significance was determined by *t*-test. ^*^p < 0.05, ^**^p < 0.01, ^***^p < 0.001, and ns: not significant.

Micro-CT 3D reconstructions (Figure 3A) confirmed the successful establishment of a periodontitis model in mice. Compared to HC, PC exhibited significantly increased distances from the cementoenamel junction to the alveolar bone crest, indicating alveolar bone resorption (Figure 3B), which negatively correlated with *A. muciniphila* abundance (Figure 3C). H&E staining revealed reduced alveolar bone height, rete peg proliferation of junctional epithelium, widening of the periodontal ligament space, and dense inflammatory infiltrates (Figure 3D). Immunohistochemical staining showed markedly higher IL-1β expression in the PC group (Figures 3D,E), reflecting local periodontal inflammation. Beyond oral inflammation, intestinal alterations were also observed. In colonic H&E sections, crypt depth was greater in HC, suggesting faster epithelial turnover, while shallow crypts in PC may indicate impaired barrier function, epithelial injury, and reduced regenerative capacity (Figures 3F,G). MUC2, a mucin secreted by goblet cells, was significantly decreased in PC (Figures 3F,H), implying degradation of the mucus layer and increased vulnerability to microbial invasion. *A. muciniphila*, which utilizes MUC2 as its sole nutrient source, was reduced in the PC, and its abundance positively correlated with MUC2 expression (Figure 3I). ZO-1, a key tight junction protein, showed reduced positive staining in PC (Figures 3F,J), though mRNA and protein levels did not differ significantly (Figure 3L), suggesting potential but subclinical barrier impairment. Similarly, no significant differences were observed in other junction proteins such as JAM3, CLAUDIN15, and OCCLUDIN (Figure 3L), indicating that epithelial integrity may not rely on a single molecule. Elevated IL-6 and TNF-*α* levels in the PC (Figure 3K) further indicated systemic inflammatory responses. Collectively, these findings suggest that periodontitis disrupts both oral and gut microenvironments, increasing intestinal susceptibility to inflammation and infection, thereby supporting the existence of a bidirectional oral-gut axis.

### 3.3 Fecal microbiota transplantation alters gut microbiota composition in periodontitis mice

To further explore the impact of distinct fecal microbiota on the gut environment of periodontitis mice, 16S rRNA sequencing revealed significant shifts in gut microbial composition post-FMT, particularly in the abundance of *A. muciniphila* (Figures 2B,C). *A. muciniphila* levels were markedly higher in the P-H, whereas both P-P and P-PBS exhibited significantly lower levels, with no difference between the two, indicating that transplantation of microbiota from healthy individuals restored *A. muciniphila* abundance, while microbiota from periodontitis patients had no such effect. Alpha diversity (Chao1 and Shannon indices) showed no significant differences across groups (Figure 2D), suggesting comparable species richness. However, principal coordinate analysis (PCoA) based on unweighted UniFrac distances demonstrated a clear separation of microbial profiles among groups (Figure 2E). Notably, P-H clustered distinctly and separately from P-P and P-PBS, highlighting the capacity of healthy donor FMT to reshape gut microbial communities. In contrast, P-P and P-PBS microbiota compositions were similar, implying that patient-derived transplants failed to modulate dysbiosis and may even harbor pro-inflammatory microbial signatures. At the genus level (Figure 2F), P-H displayed increased abundance of beneficial genera such as *Akkermansia*, *Lactobacillus*, and *Eubacterium-F*, while pro-inflammatory taxa including *Desulfovibrio* and *Clostridium* were enriched in the P-P group. Phylogenetic heatmap (Figure 2G) and cladogram (Figure 2H) analyses confirmed the enrichment of probiotic taxa in P-H and inflammatory taxa in P-P. Interestingly, fasting blood glucose levels were significantly lower in the P-H, and moderately reduced in the P-P compared to P-PBS (Figure 2I), suggesting systemic metabolic benefits associated with healthy donor microbiota. Collectively, these findings support the potential of healthy FMT to re-establish gut microbial homeostasis and offer a novel adjunctive strategy for managing periodontitis.

### 3.4 Correlation between bone-related parameters and *Akkermansia muciniphila* expression following fecal microbiota transplantation in periodontitis mice

**Figure 4:**
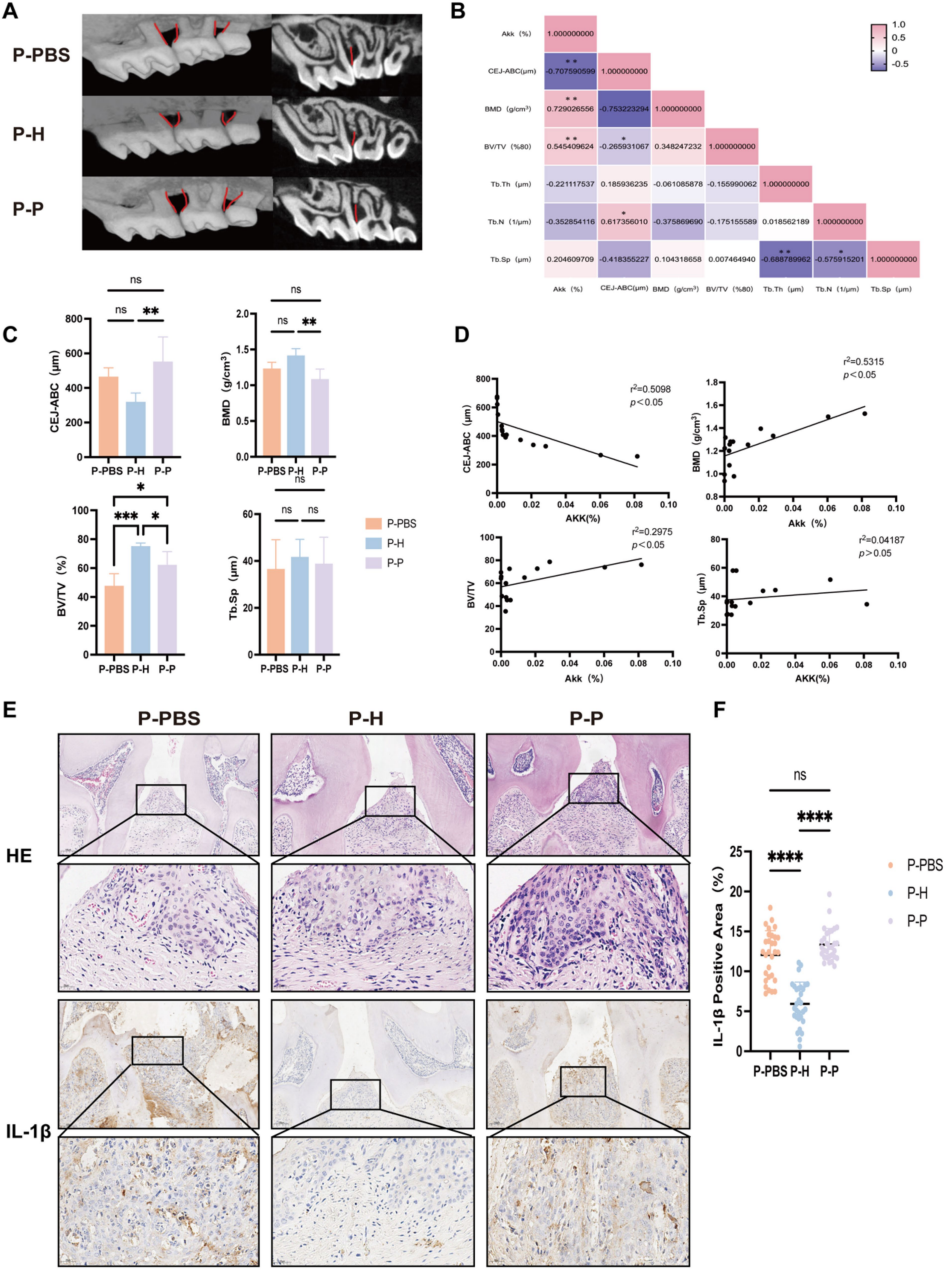
Changes in maxillary bone-related parameters in periodontitis mice following gavage with PBS, fecal microbiota from healthy individuals, or from severe periodontitis patients. **(A)** Micro-CT three-dimensional reconstruction images of the maxillary bone in periodontitis mice following different gavage treatments. **(B)** Heatmap showing correlation analysis between the relative abundance of *A. muciniphila* (%) and various bone-related parameters. **(C)** Quantitative comparison of maxillary bone parameters, including the distance between the cementoenamel junction and CEJ-ABC, BMD, BV/TV, and Tb. Sp. **(D)** Correlation analysis between *A. muciniphila* (%) and CEJ-ABC, BMD, BV/TV, and Tb. Sp. **(E)** Representative histological sections of the maxillary bone stained with hematoxylin-eosin (HE) and IL-1β immunohistochemistry. **(F)** Quantification of IL-1β positive expression rate. Data are presented as mean ± standard deviation (SD). Statistical significance was determined using one-way ANOVA. ^*^p < 0.05, ^**^p < 0.01, ^***^p < 0.001, and ns: not significant.

To investigate whether different FMT interventions affect the progression of periodontitis in mice, we performed micro-CT and immunohistochemical analyses on the maxillary bone of treated mice. Compared with the P-PBS and P-P, the P-H exhibited attenuated alveolar bone resorption, as evidenced by a significantly reduced distance between the cementoenamel junction and alveolar bone crest (CEJ-ABC) (Figures 4A,C). Furthermore, bone mineral density (BMD) was significantly increased, and bone volume fraction (BV/TV) was markedly elevated in the P-H group (Figures 4A,C). No significant difference was observed in trabecular separation (Tb. Sp) among the groups (Figures 4A,C). Correlation analysis revealed that CEJ-ABC distance, BMD, and BV/TV were associated with the abundance of *A. muciniphila* in the gut (Figure 4D). Specifically, BMD and BV/TV showed a positive correlation with *A. muciniphila* levels, whereas CEJ-ABC distance was negatively correlated. In addition, a heatmap of correlation coefficients (Figure 4B) showed that alveolar bone resorption was negatively correlated with BV/TV and positively correlated with trabecular number (Tb. N). Tb. Sp exhibited a negative correlation with both Tb. Th and Tb. N. Histological analysis using H&E staining showed more severe inflammatory infiltration and alveolar bone loss in the P-PBS and P-P groups compared to the P-H group (Figure 4E). Immunohistochemical staining for IL-1β demonstrated that the P-H group had a significantly lower positive expression rate of this pro-inflammatory cytokine than the P-PBS and P-P groups, with no significant difference observed between the latter two groups (Figures 4E,F).

### 3.5 Altered expression of intestinal tight junction proteins and inflammatory cytokines in periodontitis mice following fecal microbiota transplantation

**Figure 5:**
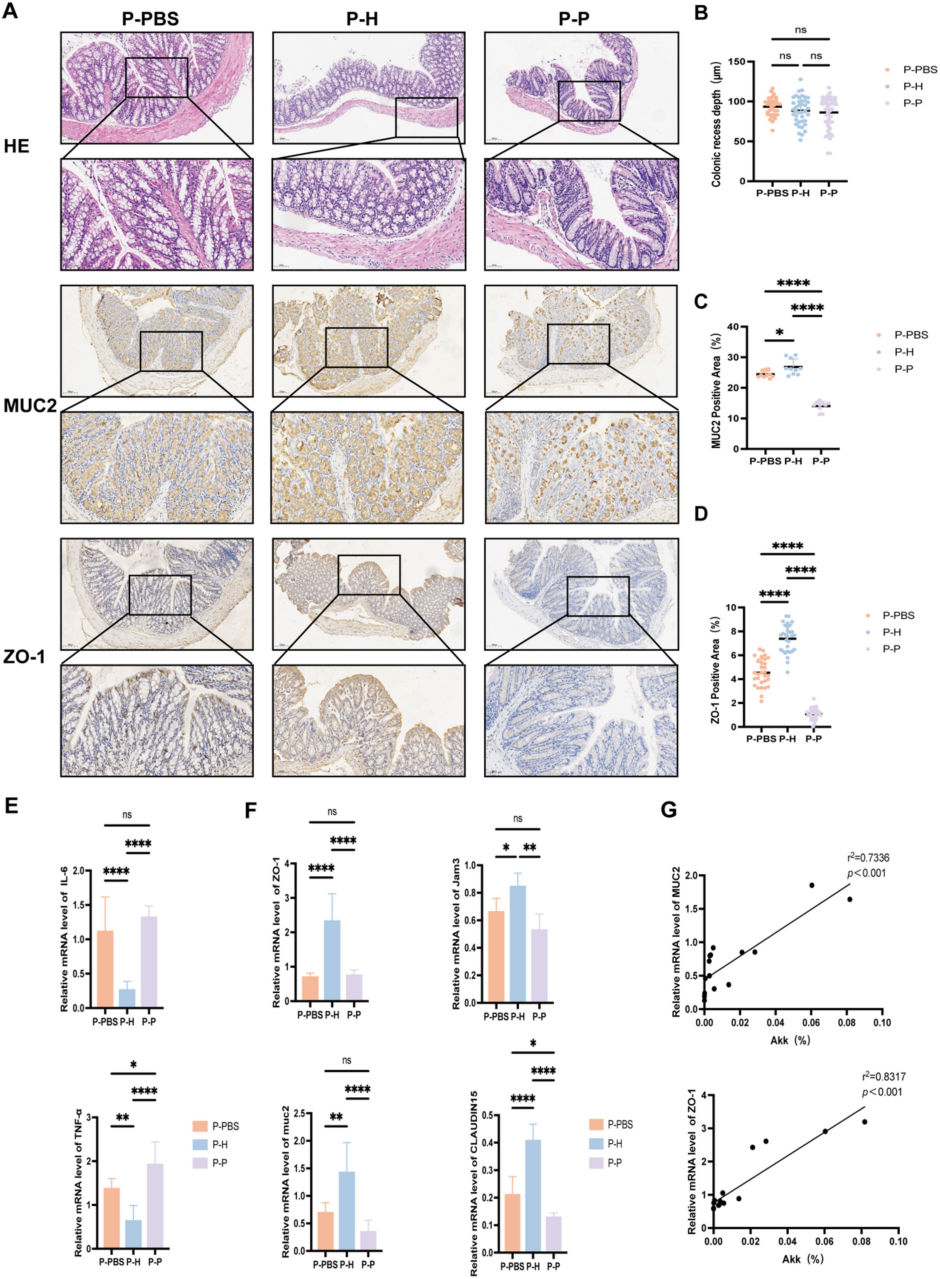
Expression of colonic proteins and inflammatory cytokines in periodontitis mice. **(A)** Representative histological images of colonic tissue sections (HE, MUC2, and ZO-1 staining) from periodontitis mice after different fecal microbiota transplantation treatments (10× and 20× magnifications). **(B)** Crypt depth measurement. **(C)** Quantification of MUC2-positive staining area (%). **(D)** Quantification of ZO-1-positive staining area (%). **(E)** mRNA expression levels of inflammatory cytokines IL-6 and TNF-α. **(F)** mRNA expression levels of tight junction-related genes ZO-1, JAM3, CLAUDIN15, and MUC2. **(G)** Correlation analysis between the relative abundance of *A. muciniphila* (%) and protein expression of MUC2 and ZO-1. Data are presented as mean ± SD. Statistical significance was determined using one-way ANOVA. ^*^p < 0.05, ^**^p < 0.01, ^***^p < 0.001, and ns: not significant.

To investigate whether FMT can improve intestinal barrier function and reduce inflammation in periodontitis mice, we further analyzed the colonic expression of tight junction proteins, inflammatory cytokines (IL-6, TNF-α), and MUC2 after different treatment conditions. Immunohistochemical analysis (Figure 5A) revealed no significant differences in colonic crypt depth among the groups (Figure 5B); however, expression levels of MUC2 and ZO-1 were significantly altered (Figures 5C,D). Specifically, mice receiving FMT from healthy donors (P-H group) exhibited significantly higher colonic expression of MUC2 and ZO-1 compared to the P-PBS and P-P groups. Quantitative PCR analysis further demonstrated that, relative to the P-PBS and P-P groups, the P-H group had significantly reduced colonic expression of pro-inflammatory cytokines IL-6 and TNF-α (Figure 5E), along with increased expression of tight junction-related genes including CLAUDIN15, JAM3, and ZO-1 (Figure 5F). In addition, the abundance of *A. muciniphila* in the gut was positively correlated with MUC2 and ZO-1 protein expression (Figure 5G). Collectively, these findings suggest that FMT from healthy individuals may enhance intestinal barrier integrity and suppress inflammatory responses in periodontitis mice, providing evidence for the potential therapeutic role of healthy donor-derived fecal microbiota in the management of periodontitis.

## 4 Discussion

Periodontitis, while primarily recognized as an oral disease, is increasingly associated with a variety of systemic conditions. Periodontal pathogens such as *Porphyromonas gingivalis* and their virulence factors including gingipains and lipopolysaccharides (LPS) are capable of activating systemic inflammatory pathways, leading to chronic low-grade inflammation in multiple organs such as blood vessels and the liver. Moreover, these pathogens can enter the bloodstream, causing bacteremia and potentially reaching the brain to impair cognitive functions (Gasmi Benahmed et al., 2022). Studies (Kozarov et al., 2005) have isolated periodontal pathogens including *Aggregatibacter actinomycetemcomitans* and *P. gingivalis* from atherosclerotic plaques, suggesting that distant colonization exacerbates infection and may contribute to organ dysfunction. Diabetic individuals are more susceptible to periodontitis, and hyperglycemia impairs periodontal tissue repair. *P. gingivalis* has been shown to directly degrade insulin receptors through its gingipains, promoting insulin resistance (Liu et al., 2024). In colitis mouse models, *P. gingivalis* aggravates intestinal inflammation by suppressing the gut microbiota-derived linoleic acid (LA) metabolic pathway and disrupting the Th17/Treg cell balance in a microbiota-dependent manner (Jia et al., 2024). A cross-sectional and longitudinal cohort study (Chen et al., 2023) reported higher blood pressure in patients with periodontitis compared to controls, and several oral species, including *Veillonella*, were found to be enriched in the gut of hypertensive patients. Additionally, oral microbiota transplantation from periodontitis patients into DSS-induced colitis mice increased anxiety-like behavior, indicating a direct effect of the oral-gut microbiome axis on host behavior (Qian et al., 2023). Recent research increasingly focuses on the bidirectional relationship of the oral-gut axis. Periodontitis has been shown to alter gut microbiota composition, particularly affecting the abundance of *A. muciniphila*, a species thought to influence the progression of periodontitis and thus may represent a novel target for adjunctive therapies.

Periodontitis perturbs the oral microbiota, leading to dysbiosis that extends to the gut (Kitamoto et al., 2020). Comparative analyses have revealed differences in gut microbiota between periodontitis patients and healthy individuals (Bao et al., 2022). While a healthy gut is typically dominated by phyla such as *Firmicutes* and *Bacteroidetes*, individuals with periodontitis exhibit decreased *α*-diversity, a reduced *Firmicutes*/*Bacteroidetes* ratio, and an increase in *Proteobacteria* and *Verrucomicrobia*. *Bacteroidetes* and *Faecalibacterium* levels are elevated in the feces of periodontitis patients, whereas *Lactobacillus* is the only enriched genus in healthy individuals. Periodontal treatment significantly reduces the abundance of pathogenic phyla, aligning microbial composition with that of healthy individuals (Baima et al., 2024). Metagenomic and metabolomic analyses (Jia et al., 2024) have shown that oral *P. gingivalis* administration increases *Bacteroidetes* while decreasing *Firmicutes*, *Verrucomicrobia*, and *Actinobacteria*. In our study, consistent with these findings, periodontitis mice demonstrated elevated *Bacteroidetes* and reduced *Firmicutes* and *Verrucomicrobia* compared to healthy controls. In healthy adults, fecal *A. muciniphila* levels are approximately 10^6^–10^8^ CFU/g. Our data showed a significant reduction of *A. muciniphila* in feces from patients with severe periodontitis, which gradually recovered following periodontal therapy. This reduction was also observed in periodontitis mice, supporting the notion that periodontitis disrupts gut microbial structure, particularly decreasing *A. muciniphila* abundance. Systemically, periodontitis induces inflammation as evidenced by elevated serum IL-6 and TNF-α levels in periodontitis mice. It also compromises intestinal barrier function, as shown by decreased expression of ZO-1 and MUC2—key components of epithelial integrity and mucin layers, respectively. *A. muciniphila* metabolizes mucins to produce monosaccharides, oligosaccharides, and short-chain fatty acids (SCFAs), which serve as energy sources for both the host and other commensal bacteria. *A. muciniphila*, a mucin-degrading gut symbiont, has garnered attention for its probiotic potential in modulating oral and gut microbial balance, enhancing barrier integrity, regulating immune responses, and mitigating systemic inflammation. In metabolic-inflammatory diseases such as diabetes and obesity, *A. muciniphila* abundance inversely correlates with disease severity (Han et al., 2025). Its beneficial effects are thought to involve restoration of mucosal thickness, production of antimicrobial peptides, and reduction of systemic inflammation. Post-bariatric surgery, *A. muciniphila* levels rebound in obese individuals, and its supplementation improves glycemic control even in non-obese patients with type 2 diabetes (Zhang et al., 2025). *A. muciniphila* promotes glucose homeostasis through its secreted protein P9, which interacts with intercellular adhesion molecule-2 (ICAM-2), and alleviates metabolic dysregulation (Yan et al., 2021). In agreement with previous findings, our study showed reduced *A. muciniphila* levels in feces of diabetic periodontitis mice. Furthermore, *A. muciniphila* abundance negatively correlated with fasting blood glucose levels. Notably, FMT from healthy donors restored *A. muciniphila* levels and significantly lowered blood glucose in periodontitis mice. Even FMT from periodontitis patients modestly reduced blood glucose compared to untreated mice, suggesting that enhancing *A. muciniphila* abundance may benefit both periodontal and metabolic health, particularly in diabetic individuals with periodontitis.

Studies have shown that probiotics can promote overall health through various mechanisms, including modulating the balance of oral and gut microbiota, enhancing barrier function, regulating immune responses, and inhibiting carcinogens (Huang et al., 2025). *A. muciniphila* has been detected in human saliva, and its oral administration is considered safe (Depommier et al., 2019). It modulates immune responses and reduces inflammation, making it a promising candidate for oral health interventions. It can inhibit *Fusobacterium nucleatum* by suppressing expression of its virulence gene *FadA*, and reduce pro-inflammatory cytokines such as IL-1β, IL-6, and TNF-*α*, thereby attenuating *F. nucleatum*-induced periodontal destruction (Song B. et al., 2023). *A. muciniphila* also counteracts *P. gingivalis*-induced alveolar bone loss by upregulating anti-inflammatory IL-10 and downregulating pro-inflammatory IL-12 (Huck et al., 2020). In obese mouse models, pasteurized *A. muciniphila* administration alleviated *P. gingivalis*-induced periodontal tissue destruction, reduced plasma TNF-α, and increased IL-10 levels (Mulhall et al., 2022). Similarly, supplementation with *A. muciniphila* or its outer membrane protein Amuc_1100 in mice promotes M2 macrophage polarization and alleviates alveolar bone destruction (Mulhall et al., 2020). Our study showed that FMT from healthy individuals increased *A. muciniphila* levels in periodontitis mice, reduced CEJ-ABC distance, and limited alveolar bone resorption. Bone-related indicators such as BMD and BV/TV were positively associated with *A. muciniphila* abundance, while IL-1β levels were reduced, further supporting its protective role against periodontal disease progression. Gram-negative bacteria secrete endotoxins such as LPS that translocate into circulation through compromised gut barriers, potentially triggering autoimmune responses (Zhou et al., 2023). *A. muciniphila* has been demonstrated to reduce serum LPS levels (Plovier et al., 2017) and improve host metabolic and immune function through specific metabolites including P9 protein (Yoon et al., 2021), outer membrane protein Amuc_1100 (Macchione et al., 2019) and short-chain fatty acids (Ioannou et al., 2025). It enhances epithelial integrity by increasing tight junction proteins (e.g., ZO-1, CLAUDIN15) and restoring mucin production. Our findings revealed that FMT from healthy donors increased intestinal *A. muciniphila*, upregulated tight junction proteins (ZO-1, CLAUDIN15, JAM3), and MUC2 expression, improved gut barrier function, and suppressed intestinal inflammation by reducing IL-6 and TNF-α levels. However, the exact molecular mechanisms underlying these effects remain to be elucidated.

However, several limitations of the present study should be considered. The limited sample size of human participants may affect the generalizability of the findings, and validation in larger cohorts is warranted. The three-month follow-up period may not be sufficient to capture long-term microbial and immunological changes following periodontal therapy. Additionally, while the ligature-induced periodontitis model in mice provides a controlled experimental setting, it does not fully mimic the complexity and chronic progression of human periodontitis. Although *A. muciniphila* was observed to be enriched following treatment and associated with improved outcomes, further studies are needed to clarify its specific role and underlying mechanisms in modulating host inflammation. The findings highlight the potential relevance of the gut-oral axis in periodontal inflammation. Modulating gut microbiota composition, particularly increasing *A. muciniphila* abundance, may represent a complementary approach in managing periodontitis and related systemic conditions. However, these insights require further mechanistic exploration and clinical translation before being considered for therapeutic application.

## 5 Conclusion

Our results demonstrate that periodontitis reduces *A. muciniphila* abundance in the gut. Restoration of *A. muciniphila* levels improves intestinal microenvironment and barrier integrity, attenuates systemic inflammation, and potentially modulates periodontitis progression. These findings provide novel insights into the therapeutic potential of *A. muciniphila* as an adjunctive strategy for managing periodontitis and associated systemic conditions.

## Data Availability

The 16S rRNA sequencing data presented in this study are publicly available. This data can be found here: https://www.ncbi.nlm.nih.gov, accession number PRJNA1313001.
