# Correction: *Akkermansia muciniphila* regulates the gut microenvironment and alleviates periodontal inflammation in mice with periodontitis

**DOI:** 10.3389/fmicb.2026.1892163

**Published:** 2026-06-16

**Authors:** Shumin Zhang, Ting Zhang, Yiwen Zhang, Chuanjin Ye, Litong Mu, Qinghui He, Tianxiang Huang, Guowei Wang, Yanan Li, Sijing Xie, Xuna Tang

**Affiliations:** 1 Nanjing Stomatological Hospital, Affiliated Hospital of Medical School, Institute of Stomatology, Nanjing University, Nanjing, China; 2 School of Food Science and Pharmaceutical Engineering, Nanjing Normal University, Nanjing, China

**Keywords:** periodontitis, *Akkermansia muciniphila*, gut microbiota, oral-gut axis, fecal microbiota transplantation

The title of this article was erroneously given as: [*Akkermansia muciniphila* regulates the gut microenvironment and alleviate periodontal inflammmation in mice with periodontitis]. The correct title of the article is [*Akkermansia muciniphila* regulates the gut microenvironment and alleviates periodontal inflammation in mice with periodontitis].

The original version of this article has been updated.

