# Supplementary material for: Akkermansia muciniphila regulates the gut microenvironment and alleviates periodontal inflammation in mice with periodontitis
Source: Front Microbiol. 2025 Sep 17;16:1643691. doi: 10.3389/fmicb.2025.1643691 (PMC12486416; doi:10.3389/fmicb.2025.1643691)
Supplement: Supplementary file 1 [file Table_1.DOCX]

**Materials and Methods**

*Human Fecal Sample Collection and Processing*

Fecal samples were collected from 16 periodontally healthy individuals and 12 patients with severe periodontitis at the Affiliated Stomatological Hospital of Nanjing University School of Medicine, with approval from the Ethics Committee of the Nanjing University School of Medicine (NJSH-2022NL-43). All participants provided informed consent prior to enrollment. Fresh stool samples were collected using sterile fecal collection devices, with approximately 3-5g of the middle portion of the stool placed into sterile tubes. The samples were labeled and mixed with 20% sterile glycerol, then rapidly frozen in liquid nitrogen and stored at -80°C until use. A portion of the samples was used for 16S rRNA sequencing, while another portion was thawed as needed, suspended in sterile PBS (200mg feces diluted to a final volume of 2mL), filtered, vortexed for 5 minutes, and centrifuged at 600×g for 5 minutes to remove insoluble matter. The resulting supernatant was used for gavage in periodontitis mice (gavage volume was approximately 10μL/g, based on the mouse body weight). The inclusion and exclusion criteria were as follows:

Table 1 Inclusion and Exclusion Criteria for Participants

| **Group** | **Inclusion Criteria** | **Exclusion Criteria** |
| --- | --- | --- |
| **Periodontally Healthy Group (H group)** | (1) Age: 25–40 years;  (2) Intact periodontal tissues: BOP < 10%, PD ≤ 3mm, no attachment loss, and no changes in gingival color, shape, or texture. | (1) History of periodontal treatment within the past year;  (2) Use of local or systemic antibiotics within the past 6 months;  (3) Systemic diseases: gastrointestinal disorders, diabetes, hypertension, cardiovascular or cerebrovascular diseases;  (4) Severe oral diseases;  (5) Infectious diseases: syphilis, HIV, etc.;  (6) Pregnant, breastfeeding, or history of miscarriage within 3 months;  (7) Smoking history. |
| **Severe Periodontitis Group (P group)** | (1) Age: 25–40 years;  (2) Diagnosis of periodontitis: Stage III or above, generalized (involving ≥30% of teeth). |  |

*Mice and Study Design*

Eight-week-old male C57BL/6J mice were purchased from Shanghai SLAC Laboratory Animal Co., Ltd., and were maintained under SPF conditions at Jiangsu Ailingfei Biotechnology Co., Ltd. All mice were acclimated for 1 week prior to the experiment. After the gavage experiments, the mice were euthanized via cervical dislocation under isoflurane anesthesia. Each group consisted of 8 mice. All animal experiments were approved by the Animal Welfare and Ethics Review Committee of Jiangsu Ailingfei Biotechnology Co., Ltd. (JSAB24021M).

Study 1: Alteration of Gut Microbial Structure in Healthy and Periodontitis Mice, investigated the changes in gut microbial structure between healthy mice and periodontitis mice.

Study 2: Fecal Microbiota Transplantation, fecal microbiota from healthy individuals and patients with severe periodontitis were transplanted into periodontitis mice to explore changes in gut microbial structure following different gavage treatments. The goal was to verify the impact of key gut microbial species on periodontitis and systemic inflammation. The study proceeded as follows:

*Study 1: Establishment of Periodontitis Model in Mice*

*A total of 16 mice were randomly assigned to 2 groups: (1) Healthy group (H); (2) Periodontitis group (P). Mice in the P group underwent ligation of the maxillary bilateral first molars to establish the periodontitis model. After 2 weeks of treatment, the model was successfully established.*

*Study 2: Fecal Microbiota Transplantation*

*A total of 24 periodontitis mice were randomly assigned to 3 groups: (1) Gavage with PBS (P-PBS); (2) Gavage with fecal microbiota from healthy individuals (P-H); (3) Gavage with fecal microbiota from periodontitis patients (P-P). All mice were administered an antibiotic mixture (ampicillin 1mg/ml, vancomycin 0.5mg/ml, neomycin 1mg/ml, metronidazole 1mg/ml) for 2 weeks to clear their gut microbiota. Gavage was performed according to the mouse body weight (200mg fecal suspension/mouse/administration), with 2-3 gavage administrations per week for 2 weeks.*
